# Continuous bladder urinary oxygen tension as a new tool to monitor medullary oxygenation in the critically ill

**DOI:** 10.1186/s13054-022-04230-7

**Published:** 2022-12-16

**Authors:** Raymond T. Hu, Yugeesh R. Lankadeva, Fumitake Yanase, Eduardo A. Osawa, Roger G. Evans, Rinaldo Bellomo

**Affiliations:** 1 Department of Anaesthesia, Austin Health, Heidelberg, VIC Australia; 2 Department of Critical Care, Melbourne Medical School, The University of Melbourne, Parkville, VIC Australia; 3 Pre-Clinical Critical Care Unit, Florey Institute of Neuroscience and Mental Health, University of Melbourne, Parkville, VIC Australia; 4 Department of Intensive Care, Austin Hospital, Heidelberg, Australia; 5 Cardiology Intensive Care Unit, DF Star Hospital, Brasília, Brazil; 6 D’Or Institute for Research and Education (IDOR), DF Star Hospital, Brasília, Brazil; 7 Cardiovascular Disease Program, Biomedicine Discovery Institute and Department of Physiology, Monash University, Clayton, VIC Australia; 8 Australian and New Zealand Intensive Care Research Centre, Monash University, Melbourne, Australia; 9 Department of Intensive Care, Royal Melbourne Hospital, Parkville, Australia

**Keywords:** Acute kidney injury, Urine oximetry, Renal medullary hypoxia, Critical care

## Abstract

Acute kidney injury (AKI) is common in the critically ill. Inadequate renal medullary tissue oxygenation has been linked to its pathogenesis. Moreover, renal medullary tissue hypoxia can be detected before biochemical evidence of AKI in large mammalian models of critical illness. This justifies medullary hypoxia as a pathophysiological biomarker for early detection of impending AKI, thereby providing an opportunity to avert its evolution. Evidence from both animal and human studies supports the view that non-invasively measured bladder urinary oxygen tension (PuO_2_) can provide a reliable estimate of renal medullary tissue oxygen tension (tPO_2_), which can only be measured invasively. Furthermore, therapies that modify medullary tPO_2_ produce corresponding changes in bladder PuO_2_. Clinical studies have shown that bladder PuO_2_ correlates with cardiac output, and that it increases in response to elevated cardiopulmonary bypass (CPB) flow and mean arterial pressure. Clinical observational studies in patients undergoing cardiac surgery involving CPB have shown that bladder PuO_2_ has prognostic value for subsequent AKI. Thus, continuous bladder PuO_2_ holds promise as a new clinical tool for monitoring the adequacy of renal medullary oxygenation, with its implications for the recognition and prevention of medullary hypoxia and thus AKI.

## Introduction

Acute kidney injury (AKI) is a frequent complication in intensive care units, affecting 30–60% of critically ill patients [1]. The role of renal haemodynamics and oxygenation in the evolution of acute renal dysfunction has been summarised by Ricksten and colleagues [2], who have particularly focused on clinical states where the parlous balance between oxygen supply and demand might most likely explain the pathogenesis of AKI, so-called ischemic AKI. Such states include sepsis, cardiac and other major surgery, congestive heart failure, after liver transplantation [2] and in the context of renal transplantation [3]. In particular, it is the outer medulla, with lesser perfusion than the cortex but high oxygen consumption within the thick ascending limb of the loop of Henle that is particularly susceptible to hypoxia [4]. Oxygen availability in the renal medulla is also attenuated by counter-current exchange of oxygen within the vasa recta [4]. Additionally, the renal medullary circulation appears not to be as tightly autoregulated as the renal cortical circulation [5]. Thus, the medulla is at particular risk of inadequate perfusion and oxygenation, particularly under pathophysiological conditions [6, 7]. Renal tissue hypoxia may in fact be a final common pathway [8, 9] or a critical event [10, 11] in the development of multiple forms of AKI. Furthermore, renal tissue hypoxia has been implicated in the transition of AKI to chronic kidney disease [12, 13].

Serum creatinine is used to estimate glomerular filtration rate and diagnose patients with AKI. Other plasma and urinary biomarkers, including neutrophil gelatinase-associated lipocalin and cystatin C, have demonstrated promise in the early detection of AKI [14]. However, they are markers of renal injury that has already occurred. Thus, any diagnosis that depends on such biomarkers may lag hours behind the initiating insult. Using large mammalian experimental models, renal medullary hypoxia has been demonstrated in two major contributors to AKI: sepsis and cardiac surgery involving cardiopulmonary bypass (CPB) [15–19]. In these experiments, probes are inserted into the tissue of the renal medulla of sheep. When sepsis-induced AKI is induced by *E. coli* resulting in reduced creatinine clearance [15, 16] or oliguria [17], a fall in renal medullary tissue oxygenation (tPO_2_) is seen. A similar fall is demonstrated upon initiation of CPB [18, 19]. Moreover, in the ovine septic AKI model, medullary tissue hypoxia precedes the development of functional deficits by several hours [16]. Accordingly, developing minimally invasive techniques for reliably assessing renal medullary tPO_2_ may provide a means for early detection of risk of AKI and for guiding therapies in order to avoid exacerbating that risk. However, it is not clinically feasible to directly monitor renal medullary tPO_2_ in patients. Our research group has proposed that continuous measurement of bladder urinary oxygen tension (PuO_2_) could provide a non-invasive surrogate measure of renal medullary tPO_2_ and thus could be an appropriate pathophysiological biomarker of AKI [10]. The rationale for this proposition is supported by the following lines of evidence (summarised in [11]): that (i) the anatomy of the renal medulla facilitates diffusion of oxygen between vasa recta and the collecting ducts, so that (ii) PuO_2_ in the renal pelvis equilibrates with renal medullary tPO_2_, (iii) that oxygen diffusion across the epithelium of the ureter and bladder only partially confounds the relationship between medullary tPO_2_ and PuO_2_ and that (iv) bladder PuO_2_ provides reliable prognostic information. A selection of studies separated into animal and human data is presented below as support for these assertions.

## Experimental studies in animals

Initial pioneering work between 1958 and 1960 focused on canine experiments, establishing that the urine in the renal pelvis and the tissue of the renal medulla were areas of low oxygen tension [20–22], with values lower than the oxygen tension within the renal vein. However, the polarographic methods for measurement of oxygen tension used at the time were subject to measurement error, with insufficient miniaturisation to always accurately determine site of measurement [23].

**Fig. 1:**
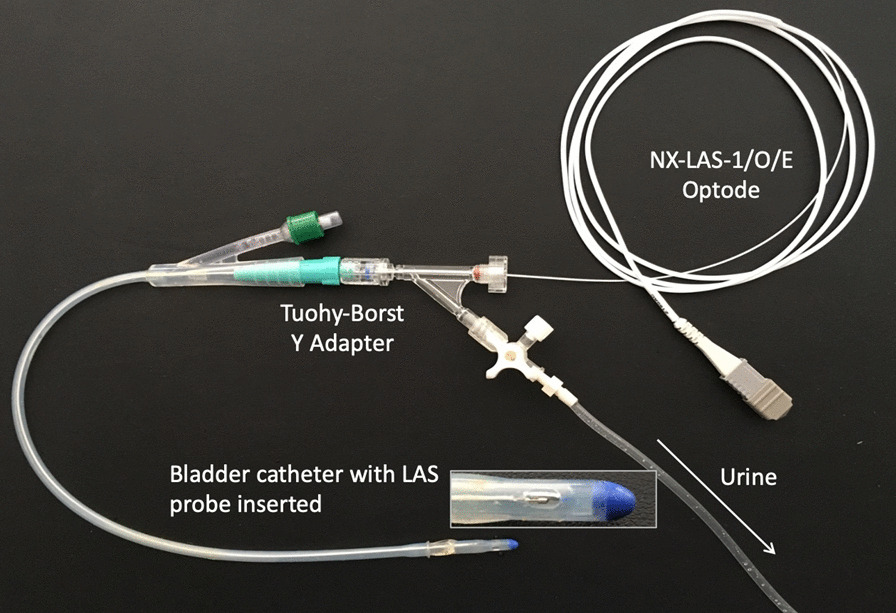
An example of a fibre optic probe (an “optode”) for fluorescence lifetime oximetry (NX-LAS-1/O/E; Oxford Optronix Ltd.; Abingdon, UK). Here, the optode has been advanced through a Foley bladder catheter to permit continuous monitoring of bladder urinary oxygen tension. Similar probes can be used to invasively measure tissue oxygen tension in various organs

More recent work has focused on using different animal models (chiefly ovine [15–17, 19, 24–27], but also murine [28, 29] and leporine [30]) to examine this area, with the use of new technology such as fibre optic fluorescence lifetime oximetry probes to continuously measure oxygen tension within urine or renal tissue (Fig. 1). This has provided insights into changes that occur during development of septic AKI, as well as during clinically relevant resuscitation strategies such as fluids, vasopressors and diuretics.

### *Prognostic utility of PuO*_*2*_* in the early detection of AKI*

**Fig. 2:**
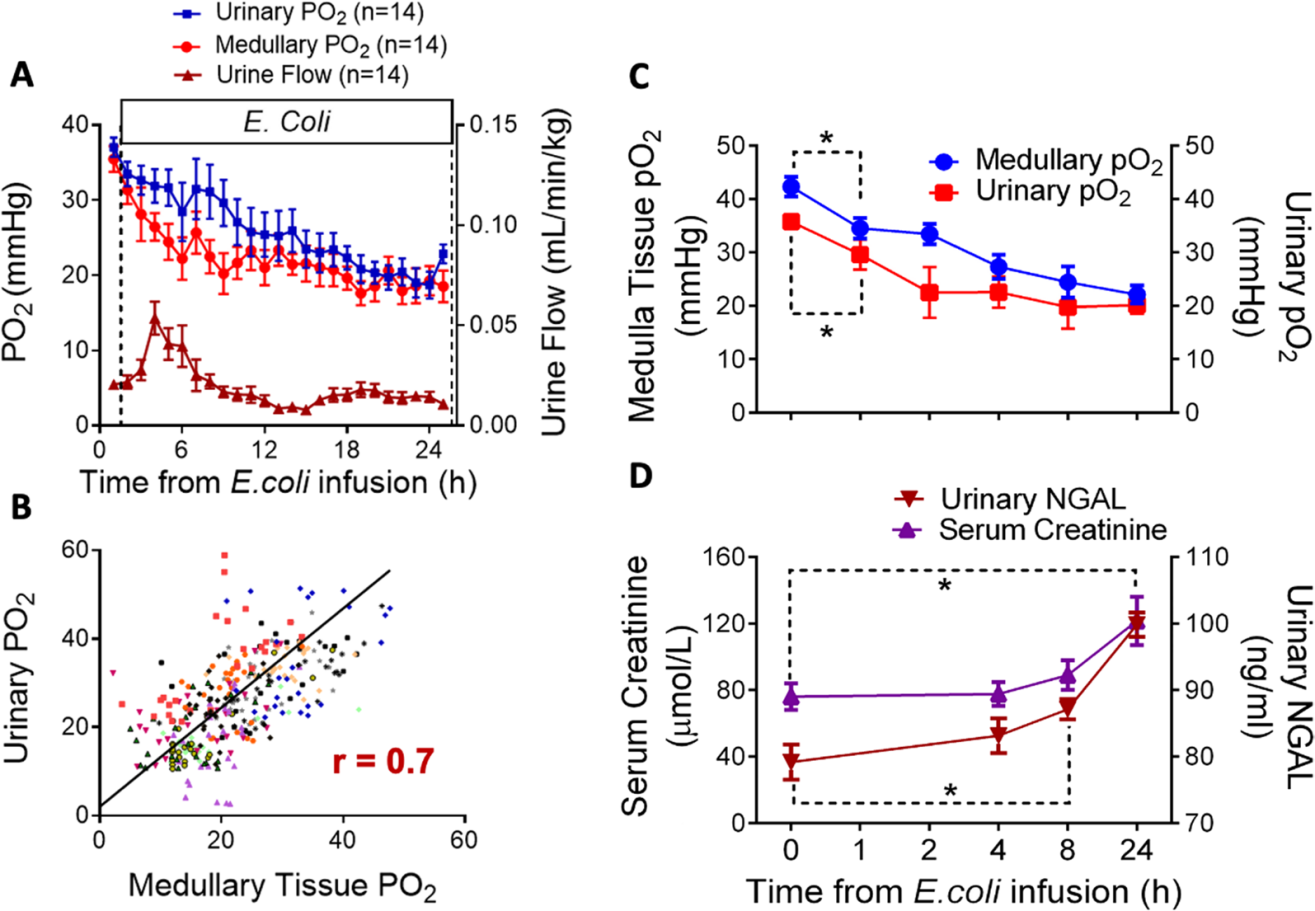
The prognostic utility of bladder urinary oxygen tension in the early detection of acute kidney injury over 24 h in Gram-negative sepsis in non-anesthetised sheep. **A** The time course of changes in renal medullary tissue PO_2_ and bladder urinary PO_2_ and urine flow over 24 h of sepsis. Urine flow is presented as absolute values corrected for kilogram of body weight. **B** Scatterplot of the relationship between medullary tissue and urinary PO_2_ (different symbols represent individual sheep). The line of best fit, determined by ordinary least-product regression analysis, had an *X* intercept of 2.4 mmHg (95% confidence interval: 0.3–4.5) and a slope of 1.02 (95% confidence interval: 0.95–1.09) (*P* < 0.001). **C** Changes in renal medullary tissue and bladder urinary PO_2_ and **D** plasma creatinine and urinary neutrophil gelatinase-associated lipocalin (NGAL) over 24 h of Gram-negative sepsis-induced acute kidney injury. *P < 0.05 indicates significant differences from pre-morbid baseline (Time 0). Figures modified from [16, 17]

In ovine sepsis, the initiation of renal medullary tissue hypoperfusion and hypoxia occurs within the first hour of Gram-negative infection followed by a progressive reduction over 24 h [15–17]. Such renal medullary microcirculatory abnormalities take place despite increased and/or preserved global renal blood flow, global renal oxygen delivery and cortical perfusion and cortical tPO_2_ in non-anesthetised septic sheep [15–17]. An early onset of renal medullary tissue hypoxia could potentially initiate and perpetuate a cycle of inflammation and oxidative/nitrosative stress that can lead to mitochondrial dysfunction, tubular cell injury and reduced kidney function [6, 31]. Reductions in renal medullary tPO_2_ were detected in ‘real time’ up to 8 to 24 h before any significant elevations in urinary neutrophil gelatinase-associated lipocalin and/or serum creatinine could be detected in septic sheep with AKI (Fig. 2C & D) [16]. Remarkably, the temporal profile and magnitude of the progressive decrease in renal medullary tPO_2_ in sepsis was closely reflected by continuously measured bladder PuO_2_ (Fig. 2A). Indeed, a positive significant correlation was detected during the development of AKI over 24 h (*r* = 0.7; *P* < 0.001; Fig. 2B) [17]. These findings strongly support the prognostic value of bladder PuO_2_ as an early physiological biomarker to evaluate risk of developing septic AKI.

### *Potential utility of PuO*_*2*_* to guide clinical interventions*

Renal medullary tPO_2_ is dominated by the energy requirements of the sodium–potassium ATPase pump required to drive tubular sodium reabsorption in the thick ascending limb of the loop of Henle. Thus, renal oxygen consumption is directly linked to the filtered load of sodium and thus glomerular filtration rate [32]. Consequently, the effects of goal-directed therapies such as fluid resuscitation, diuretics and vasopressors on renal medullary tPO_2_ reflect a complex interaction between their effects on renal and intra-renal perfusion and tubular function. For example, given that any therapy that increases glomerular filtration rate would be expected to increase renal oxygen consumption, this would, in turn, potentially promote renal tissue hypoxia. Conversely, furosemide treatment (which inhibits tubular sodium reabsorption) or fluid resuscitation was found to reverse medullary tissue hypoxia in ovine septic AKI back to healthy physiological levels [25, 26]. On the other hand, the actions of various vasoactive drugs to restore blood pressure were not universally favourable for renal medullary tissue perfusion and tPO_2_. Norepinephrine further worsened renal medullary tissue hypoperfusion and hypoxia, an undesirable effect; but this was not seen when vasopressin was used as primary vasopressor [17, 24]. Angiotensin II was found to induce minimal changes to medullary tPO_2_ in ovine septic AKI [16] but was also found to reduce medullary tPO_2_ in healthy sheep [24]. These varied effects are best understood by accounting for the myriad of factors that could affect medullary blood flow and medullary oxygen consumption, such as differential receptor occupancy of pre-glomerular (afferent) and post-glomerular (efferent) arterioles, changes in oxygen consumption due to alterations in sodium reabsorption accompanying changes in GFR or changes in microcirculatory shunting of oxygen within the counter-current exchange arrangement of vessels [24]. At a macro-circulatory level, when renal blood flow was increased in sheep undergoing CPB by increasing global perfusion (i.e. increasing systemic pump flow), increasing renal perfusion by vasopressor support with metaraminol or both [18, 33], medullary tPO_2_ also increased.

Despite the variable effects of various resuscitative measures on medullary tPO_2_, they all appear to mirror changes in bladder PuO_2_ in sheep (e.g. during resuscitation with fluids [25], norepinephrine [17] and angiotensin II [16]; as well as during administration of furosemide [26]). Similarly, when two pharmacological agents that reduced medullary tPO_2_ (vasopressin V_1-_receptor activation or blockade of nitric oxide synthesis) were administered to anesthetised rabbits, the changes in bladder PuO_2_, measured using the same technique as used in sheep, mimicked changes in medullary tPO_2_ [30]. Further evidence comes from a post-hoc analysis of experimental studies in septic sheep breathing room air, where it was demonstrated that bladder PuO_2_ varied linearly with medullary tPO_2_ under normoxic or slightly hypoxic conditions [34].

Together, these preclinical observations indicate that measurement of bladder PuO_2_ may be a clinically feasible technique to estimate renal medullary tPO_2_, and therefore vulnerability towards developing AKI, as well as a tool to gauge the impact of resuscitation strategies on medullary tPO_2_.

### Clinical studies in humans

Although PuO_2_ in humans had been reported previously (e.g. [21, 35]), the utility of urine oximetry in humans was only properly investigated in a small series of studies by Leonhardt and colleagues between 1963 and 1965 [36–39]. This early exploration of its utility occurred concurrently alongside animal work in dogs, with early postulation that there must exist an equilibrium between the oxygen tension in the collecting ducts and the peritubular capillary blood given the permeable nature of the epithelium of the collecting ducts [35]. This series of studies was performed amongst patients undergoing urological procedures to enable measurement of both pelvic ureteric PuO_2_ and sometimes renal tissue PO_2_, together with patients with an indwelling urethral catheter for measurement of bladder PuO_2_. In such patients, Leonhardt and colleagues found good correlation between pelvic ureteric PuO_2_ and medullary tPO_2_ [36] as well as a relationship between pelvic ureteric PuO_2_ and bladder PuO_2_ [37]. Furthermore, pelvic ureteric PuO_2_ increased with hydration, although the response time was delayed in patients with reduced renal perfusion (e.g. sepsis or shock) [38], whilst hypertonic saline infusion reduced pelvic ureteric PuO_2_, presumably due to the increased medullary oxygen consumption associated with salt loading [39]. Both pelvic PuO_2_ and bladder PuO_2_ were reported to rise with increasing inspired fraction of oxygen, although the response was sluggish in disease states such as pyelonephritis or azotemia [38]. Additionally, the investigators reported that PuO_2_ was altered with agents that affect sympathetic tone, in a manner independent of urine flow [37]. Leonhardt and colleagues proposed that the oxygen tension in urine could be used as an indication of medullary oxygenation and that the response time of PuO_2_ to changes in FiO_2_ could be an index of medullary blood flow at periods of stable oxygen consumption, with a slower response time indicating poorer medullary blood flow, in a manner analogous to an indicator dilution technique for determining flow [36].

Continued reports of the use of PuO_2_ largely focused on measuring effects of various interventions on bladder PuO_2_, given the ease of sampling. These included demonstration of the reduction in PuO_2_ with sevoflurane and isoflurane [40], and the improvement in PuO_2_ with the renal vasodilatory effects of dopamine, prostaglandin E_1_ and fenoldopam [41, 42]. Dexmedetomidine was observed to decreased bladder PuO_2_ in critically ill patients, which was attributed to attenuation of cardiac output and thus renal blood flow and medullary perfusion [43]. Furosemide was reported to decrease ureteric PuO_2_ in otherwise well patients after relief of urologic stones [44], but was observed to increase bladder PuO_2_ in septic patients [45]. Whilst the latter response is consistent with reductions in medullary oxygen consumption observed in animal models [26] and humans [46, 47], furosemide has also been associated with a reduction in medullary blood flow in animals [48, 49], which could explain the former response.

Studies of the effects of non-pharmacological interventions on bladder PuO_2_ have included examination of the effects of red cell transfusion and the relationship between bladder PuO_2_ and cardiac output. In a small study involving eight patients, red cell transfusion was seen to increase bladder PuO_2_ as measured by a blood gas analyser [50]. Additionally, amongst 60 patients with a recent myocardial infarction or unstable angina, bladder PuO_2_ and cardiac output (as measurement by Swan-Ganz catheter) were positively correlated [51]. These imply that global oxygen delivery (DO_2_) is relevant for determining PuO_2_.

**Fig. 3:**
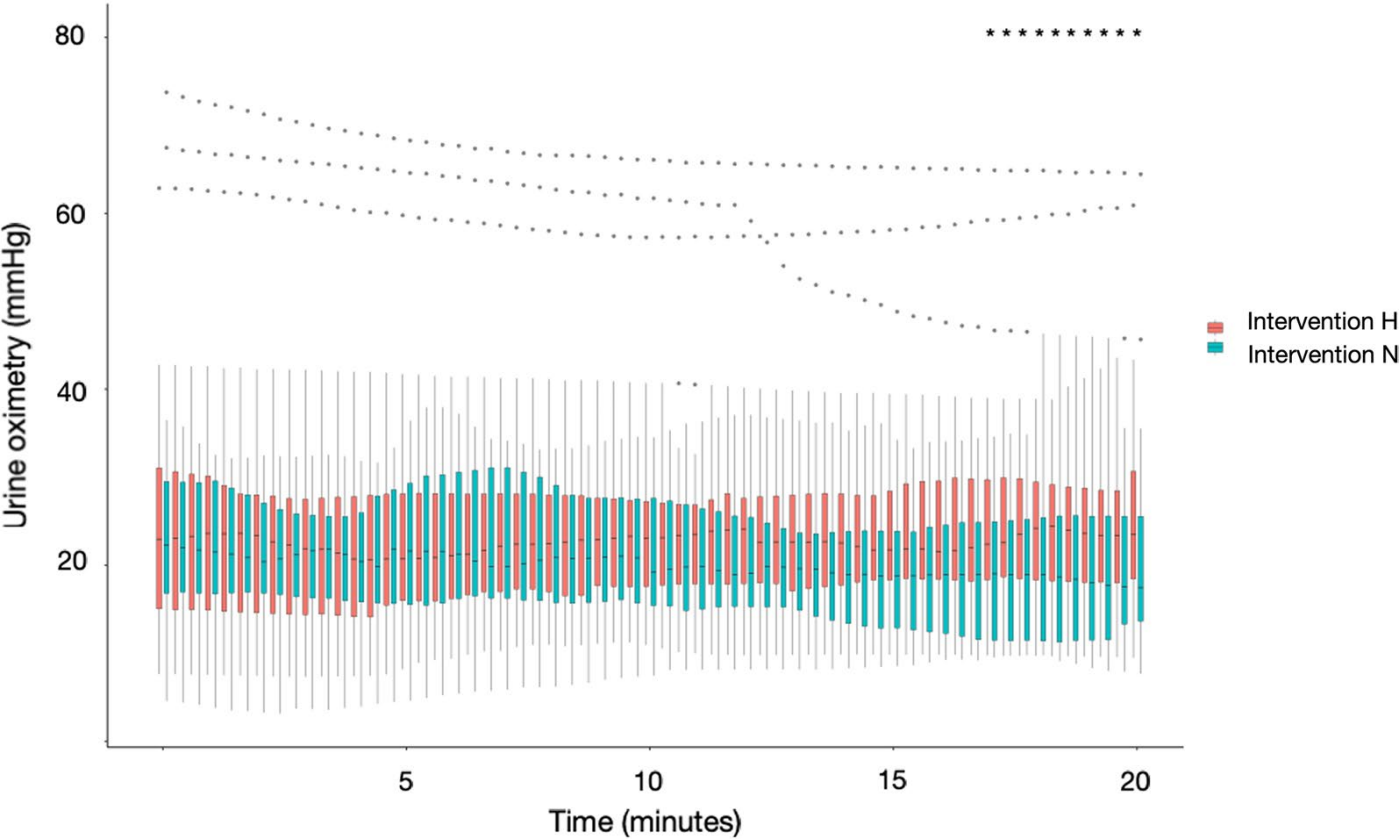
Urine oxygen tension (PuO_2_) across time during Intervention H (target pump flow of 3.0 L/min/m^2^ and mean arterial pressure (MAP) of 80 mmHg) and Intervention N (target pump flow of 2.4 L/min/m^2^ and MAP of 65 mmHg). Each box represents the interquartile range (IQR) for the 20-secondly median values across patients, with lines out to $\pm$ 1.5 $\times$ IQR. Dots represent outliers. Mixed model for repeated measures showed statistical significance for group and time interaction (*P* < 0.001). Differences in PuO_2_ between the intervention groups, adjusted for repeat measurements, showed statistically significance after approximately 17 min (marked *). Reproduced from Hu et al. [59] with permission

The association between DO_2_ and PuO_2_ or kidney outcomes has been further explored in patients undergoing CPB. During CPB, it is notable that increasing pump flow results in a more favourable renal oxygen supply relative to demand, with a lower oxygen extraction ratio [52], which is consistent with the observation that renal blood flow is directly proportional to systemic flow during CPB, implying a loss of renal autoregulatory capacity [53]. In a multicentre randomised controlled study that was terminated after interim analysis of 350 randomised patients (out of a target sample size of 700 patients), a goal-directed strategy aiming for DO_2_ > 280 ml/min/m^2^ was found to result in fewer patients developing Acute kidney Injury Network (AKIN) Stage 1 compared with usual care (relative risk 0.45, 95% confidence interval 0.25–0.83, *P* = 0.01) [54]. Furthermore, in a randomised controlled crossover trial amongst 20 patients, bladder PuO_2_ was successfully raised in response to higher CPB pump flow (and therefore systemic DO_2_) and mean arterial pressure (MAP) compared with normal CPB pump flow and MAP (achieving median [interquartile range] DO_2_ of 409 [374, 441] vs. 340 [297, 379] ml/min/m^2^ respectively) [55]. This demonstrated a successful dynamic manoeuvre to increase bladder PuO_2_ during CPB with increasing pump flow and MAP, with a response time of 17 min (Fig. 3). Notably, this higher systemic DO_2_ was achieved by targeting a CPB pump flow of 3.0 L/min/m^2^ and MAP of 80 mmHg compared with the control interval with target CPB pump flow of 2.4 L/min/m^2^ and MAP of 65 mmHg. This suggests that bladder PuO_2_ has the potential to guide management for optimising renal medullary tPO_2_ during cardiac surgery and that higher target CPB pump flow and MAP than traditionally used may be required for improving medullary oxygenation during bypass.

Whilst it is possible that the concomitant increase in renal perfusion or cortical perfusion with increased systemic DO_2_ is at least somewhat contributory to PuO_2_ (or improved kidney outcomes), the preceding discussions describing the association of PuO_2_ with medullary tPO_2_ in both animal and human studies suggest that it is medullary perfusion (and thereby oxygenation) that is the key variable that is altered. This is particularly relevant because the medullary circulation is poorly autoregulated and is determined by factors that are distinct from cortical or global renal perfusion [5].

Further support for the association between renal medullary tissue hypoxia and AKI comes from observational studies involving patients undergoing cardiac surgery requiring CPB that examined the association of bladder PuO_2_ and AKI [56–59]. In these studies, a reduction in PuO_2_ was observed upon initiation of CPB, and a slower rate of rise of PuO_2_ after weaning from bypass [56] or a lower mean PuO_2_ in the post bypass period [58] or a lower nadir and longer duration of low PuO_2_ values [57] were associated with AKI. Notably, there were no statistically significant differences in cardiac index between groups that did or did not develop AKI. However, measurement of PuO_2_ using a blood gas analyser six hours after admission to ICU had modest discrimination in detecting patients who later developed AKI [59].

## Limitations

Whilst bladder PuO2 is more convenient and less invasive for approximating medullary tPO2 than ureteric measurement, potential confounders abound. These include oxygen from the presence of blood in urine, which would be expected to contaminate samples with arterialised oxygen tension [60]. This is nevertheless easy to identify. More subtle sources of bias in bladder PuO2 measures include the presence of substances that can alter oxygen within the bladder, such as the microbiota [61] or ascorbic acid [21]. It is also well established that oxygen consumption can be observed in urine in a static state [35, 62]. Therefore, it becomes imperative that PuO2 should be obtained in the fasted state [11] and that continuous rather than static measurements of bladder PuO2 be used, ensuring that urine flow does not stop.

Bladder PuO_2_ measurements can also be altered by the diffusion of oxygen between urine and the wall of the bladder and ureter, or exposure of urine to atmospheric oxygen [34]. The uncertainty created by these confounding factors can be minimised in two ways: (i) by measuring bladder PuO_2_ in a way that minimises the opportunity for diffusion of oxygen from these sources, and (ii) through use of computational models to estimate the magnitude of this diffusion and correct for it.

Bladder PuO_2_ can be measured continuously using a fibre optic probe inserted into the bladder catheter (Fig. 1) so that its sensing tip lies within the bladder [45, 55, 57], or in the urine line external to the bladder, either using a polarographic electrode [56] or fibre optic probe [58]. Sampling external to the bladder has potential advantages in avoidance of risks associated with insertion of a probe within the bladder and potentially allowing re-use of sensors. However, it also risks the possibility of further confounding from diffusion of oxygen between the urine and the wall of the Foley catheter. Consistent with this possibility, in studies in humans, urinary PO_2_ measured in the urine line external to the bladder (38–107 mmHg) [56, 58] was higher than that measured within the bladder (26–66 mmHg) [57]. However, no direct comparison is available to determine the extent of this confounding.

Urinary PO_2_ can also potentially be measured by collecting urine from a Foley catheter for analysis in a blood gas machine [50, 51, 59]. However, when measured in this way there is the potential for oxygen to diffuse from the atmosphere (PO2 159 mmHg at sea level) to the urine sample. Consistent with this proposition, reported mean levels of urinary PO_2_ in humans using this approach [50, 59, 63–65] were markedly higher (80–153 mmHg) than those reported from studies in which urinary PO_2_ was measured in the bladder [57] (26–66 mmHg) or in the urine line external to the bladder [56, 58] (38–107 mmHg).

**Fig. 4:**
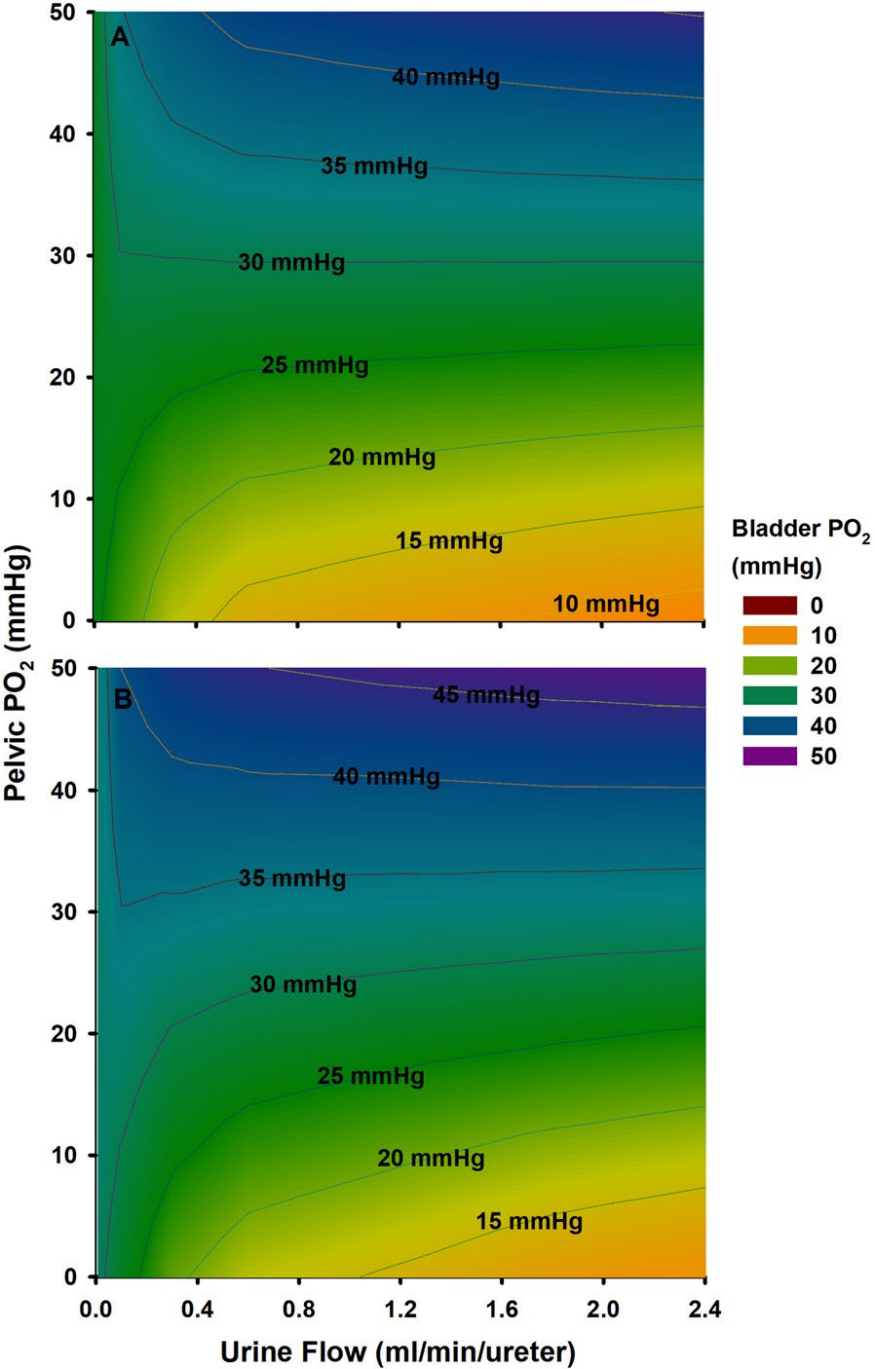
Contour plot of urine flow, bladder PuO_2_ and pelvic PuO_2_ under modelling conditions of **A** normoxia (PaO_2_ = 90 mmHg) and **B** hyperoxia (PaO_2_ = 300 mmHg) and other assumptions as per Lee et al. [75]. Figure reproduced with permission

The rate of oxygen diffusion between the urine and the walls of the ureter or bladder is driven by the gradient in PO_2_ between interfacing compartments. Bladder tPO_2_ has been shown to correlate with renal blood flow in animal studies [66, 67] although confounders to this relationship exist at the lower end of the autoregulatory limit [68], and the relationship is uncertain in the context of sepsis [69–72], and vasopressors [73, 74]. Whilst bladder tPO_2_ could contaminate bladder PuO_2_ measurement, it appears that the oxygen diffusion between bladder tPO_2_ and bladder PuO_2_ contributes little to final bladder PuO_2_ value, which is chiefly influenced by the PuO_2_ of urine entering the bladder from the ureter [30]. This ureteric PuO_2_ value is in turn determined by the time available for oxygen diffusion between ureteric wall and the urine (a function of the transit time of each bolus of urine and the bolus volume) [75]. Consequently, the major confounders of the relationship between renal medullary (or pelvic ureteric) PuO_2_ and bladder PuO_2_ appear to be the patient’s systemic oxygenation and their rate of urine flow. Thus, as urine flow becomes greater, the error in the estimate of medullary PO_2_ generated by measurement of urinary PO_2_ becomes less. In contrast, as urine flow becomes less and a patient becomes progressively hyperoxemic or hypoxemic, the error becomes greater [75]. Therefore, in a clinical setting, estimating medullary oxygenation directly from measurement of urinary PO_2_ is probably most useful when patients have a normal or high urine flow. But even when such conditions are not entirely met, computational models can account for variations in urine flow and systemic oxygenation. The most sophisticated model developed to date predicts the existence of a family of linear relationships between bladder PuO_2_ and pelvic ureteric PuO_2_ for any given set of input conditions of urine flow and systemic arterial PO_2_ in human patients (Fig. 4) [75]. Thus, it should be technically possible to predict renal medullary tPO_2_ in real time based on continuous measurement of bladder PuO_2_, provided accurate and real-time measurements of urine flow and arterial PO_2_ are available.

Finally, the implications of disease states of the kidney for measurements of bladder PuO2 remain to be fully elucidated, as recent clinical studies have been performed largely on patients without established kidney disease [55–58]. Furthermore, an elevated ureteric PuO_2_ has been noted in established hydronephrosis and could also occur with renal cysts, due to medullary thinning and the influence of renal cortical oxygenation on measurements [38]. Nevertheless, it remains possible that trends in bladder PuO_2_ could still be useful as a reflection of the adequacy of medullary oxygenation.

## Conclusion

AKI is common in critical care units, and attenuating its severity or even preventing its development is a key therapeutic target. There is increasing evidence that the inadequacy of renal medullary oxygenation is linked with its development. Furthermore, medullary tissue hypoxia appears to precede other evidence of AKI. Continuous measurements of bladder PuO_2_ promise to provide a non-invasive method for estimating medullary tPO_2_ and have prognostic value. Furthermore, it is a physiological variable that can be manipulated by pharmacological and other non-pharmacological interventions, making it an ideal candidate for targeted therapies to maintain or restore its value and thereby protect the kidney from injury. Sources of error or confounding variables remain a challenge, although many of these can be overcome by appropriate sampling and computational modelling. Harnessing the value of continuous bladder PuO_2_ monitoring appears promising in critical care environments.

## Data Availability

All data described in this manuscript are derived from published articles and have been appropriately referenced throughout.
